# Non-linear QED approach for betatron radiation in a laser wakefield accelerator

**DOI:** 10.1038/s41598-023-50030-6

**Published:** 2024-01-05

**Authors:** J. F. Ong, A. C. Berceanu, A. Grigoriadis, G. Andrianaki, V. Dimitriou, M. Tatarakis, N. A. Papadogiannis, E. P. Benis

**Affiliations:** 1 Extreme Light Infrastructure - Nuclear Physics (ELI-NP), “Horia Hulubei” National Institute for Physics and Nuclear Engineering (IFIN-HH), 30 Reactorului Street, 077125 Bucharest-Măgurele, RO Romania; 2 Institute of Plasma Physics and Lasers, University Research and Innovation Centre, Hellenic Mediterranean University, 74100 Rethimno, Crete, Greece; 3 Department of Physics, University of Ioannina, 45110 Ioannina, Greece; 4 School of Production Engineering and Management, Technical University of Crete, 73100 Chania, Greece; 5 Physical Acoustics and Optoacoustics Laboratory, Department of Music Technology and Acoustics, Hellenic Mediterranean University, 74100 Rethimnon, Greece; 6 Department of Electronic Engineering, Hellenic Mediterranean University, 73133 Chania, Greece

**Keywords:** Plasma-based accelerators, Physics, Laser-produced plasmas

## Abstract

Laser plasma-based accelerators provide an excellent source of collimated, bright, and adequately coherent betatron-type x-ray pulses with potential applications in science and industry. So far the laser plasma-based betatron radiation has been described within the concept of classical Liénard–Wiechert potentials incorporated in particle-in-cell simulations, a computing power-demanding approach, especially for the case of multi-petawatt lasers. In this work, we describe the laser plasma-based generation of betatron radiation at the most fundamental level of quantum mechanics. In our approach, photon emission from the relativistic electrons in the plasma bubble is described within a nonlinear quantum electrodynamics (QED) framework. The reported QED-based betatron radiation results are in excellent agreement with similar results using Liénard–Wiechert potentials, as well as in very good agreement with betatron radiation measurements, obtained with multi-10-TW lasers interacting with He and multielectron N$_2$ gas targets. Furthermore, our QED approach results in a dramatic reduction of the computational runtime demands, making it a favorable tool for designing betatron radiation experiments, especially in multi-petawatt laser facilities.

## Introduction

The generation of relativistic electron beams when ultra-strong laser pulses interact with plasma has been an ongoing and fruitful research topic ever since its unambiguous demonstration^1–3^. The formation and dynamics of the relativistic electron beam, in energies ranging from MeV to GeV, is elegantly described by the laser wakefield acceleration (LWFA) mechanism. A plasma bubble is initially formed by the leading edge of the laser pulse, supporting electric potentials as high as GV, in which plasma electrons are injected, and consequently accelerated to relativistic energies. LWFA accelerators are capable of sustaining a few orders of magnitude larger acceleration gradients over conventional accelerators, a fact that calls for their potential usage in science and industry as well as for the necessity of a deeper understanding and controlling of the dynamics and processes involved.

Electron acceleration under LWFA conditions is accompanied by betatron-type x-ray radiation due to the wiggling trajectories, similar to the betatron motion, that the electrons follow inside the plasma bubble. X-ray emission from the electron betatron motion in the LWFA plasma wake offers a collimated, bright, and adequately coherent radiation source suitable for many applications^4–10^, with emphasis recently given in the three-dimensional (3D) medical imaging^11,12^. Due to the complexity of the highly nonlinear plasma dynamics in LWFA and related betatron radiation, its understanding relies mainly on computer power demanding particle-in-cell (PIC) simulations^13–17^. Specifically, so far the calculation of the spectral radiation of betatron motion has been described within the concept of Liénard–Wiechert potentials, either with post-processing or in situ calculations^7,18–24^, which are both computationally high demanding approximations.

An alternative path for describing photon emission from an electron in a strong background electromagnetic field is within the concept of nonlinear quantum electrodynamics (QED). In this case, the electron trajectory is treated classically while the photons are emitted discretely according to the photon emission probability rate formulated in the constant cross-field approximation^25–27^. The direction of photon emission is the same as the electron momentum and results in synchrotron-like radiation, with electron recoils or radiation reactions incorporated^28,29^. Such an approach has been implemented in most modern PIC codes that describe plasma dynamics and related phenomena^30–33^, and is widely used in simulations of $\gamma$-ray production for nuclear applications^34–37^. However, its applicability to the description of LWFA x-ray betatron radiation has not attracted the interest of the scientific community yet.

Here, we demonstrate for the first time how the nonlinear QED approach, operating in the classical regime, can be applied to the description of betatron radiation in LWFA electron acceleration. The results on betatron radiation of our QED approach are not only in excellent agreement with similar betatron radiation calculations using Liénard–Wiechert potentials but also agree well with betatron radiation measurements, involving He and multielectron N$_2$ gas targets. In addition, the proposed method reduces dramatically the computational runtime compared to the commonly used Liénard–Wiechert approach, making it favorable for future betatron studies. Thus, aside from the interest in the proposed QED description of LWFA betatron radiation, we expect it will attract the interest of the plasma physics community at large, as deserves.

## Results

The QED emission spectrum reduces to the classical synchrotron radiation when the electron recoil is negligible and the energy of an emitted photon is much smaller than the electron energy, i.e., $\xi = \hbar \omega /(\gamma mc^2)\ll 1$. However, the photon emission probability, $\textrm{Pr}(\xi )$ exhibits an infrared divergence at $\xi =0$, i.e.,

1 $$\begin{aligned} \text {Pr}(\xi \simeq 0) \approx 0.52 \times \Delta t \times \frac{\alpha mc^2}{\hbar }\times \frac{1}{\gamma } \left( \frac{\chi }{\xi }\right) ^{2/3} > 1, \end{aligned}$$

where $\chi$ is the Lorentz invariant parameter that determines the transition between classical and quantum description, $\Delta t$ is the simulation time step, $\alpha =\frac{1}{137}$ is the fine-structure constant, *m* is electron rest mass, *c* is the speed of light in vacuum, and $\hbar$ is the reduced Planck constant. The event generator underestimates the rate of photon emission at $\xi \simeq 0$, which is the case for betatron radiation. To solve this problem in its generality we have applied the QED-*modified* event generator, described in section “Methods”, which eliminates the infrared divergence, the necessity for a low-energy cutoff and allows for modelling the entire range of incoherent emission spectrum^26^.

**Figure 1:**
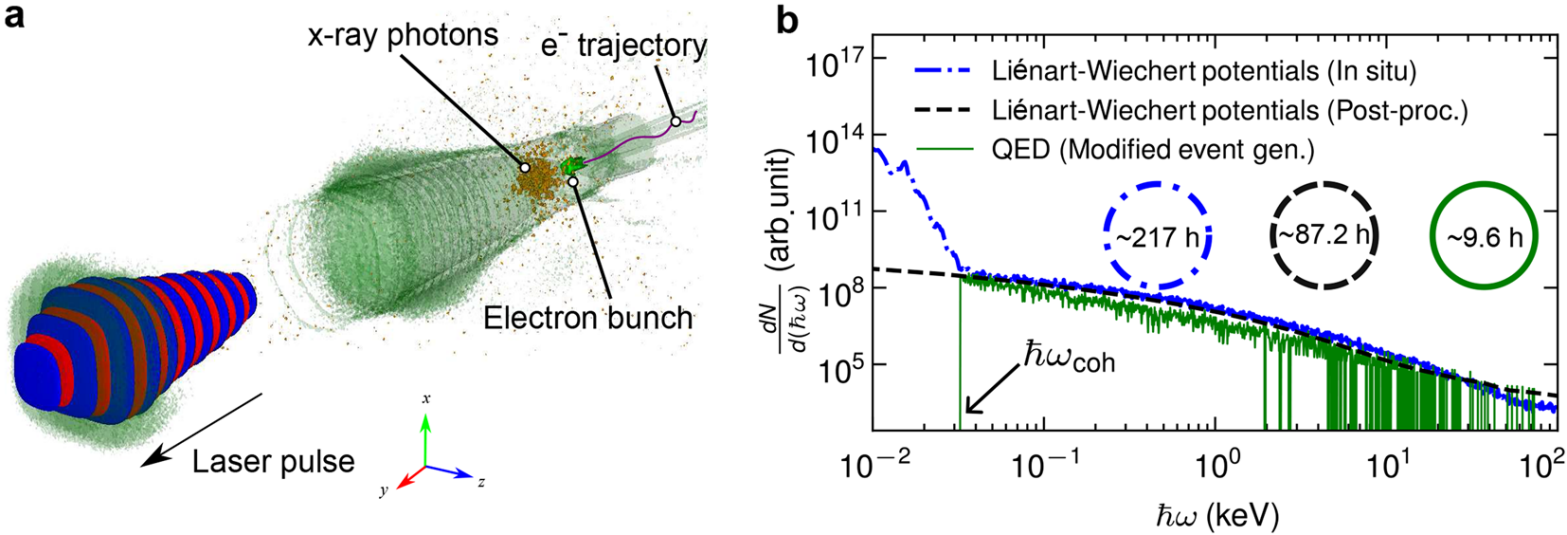
picongpu simulations of laser-driven betatron radiation. (**a**) Formation of the plasma bubble by the interaction of an intense laser pulse with helium plasma and the corresponding emission of x-ray betatron photons. The electron trajectory represents the classical oscillatory betatron motion. The laser pulse is contour-plotted by red-blue iso-surfaces, while the plasma bubble is plotted by green iso-surfaces. (**b**) Betatron x-ray spectra were calculated with Liénard–Wiechert potentials using in situ synthetic radiation diagnostic (blue dashed-dotted line) and post-processing radiation diagnostic (black dashed line). The green solid line is the spectrum calculated using the QED approach with the modified event generator in the classical limit. The transition of coherent and incoherent radiation is at $\hbar \omega _{\textrm{coh}}$. The x-ray photons emission is statistically low at $E>1\,\textrm{keV}$. The corresponding computational runtime for the three simulations is also depicted, indicating the dramatic reduction in the computational demands for the QED approach.

In Fig. 1a, a snapshot of the 3D LWFA acceleration mechanism and the emission of the x-ray betatron radiation, obtained for the experimental conditions described in section “Methods”, is illustrated. The intense laser pulse excites a nonlinear plasma wave by pushing all electrons aside and forming a spherical ion cavity termed bubble. The laser pulse is contour-plotted by red-blue iso-surfaces, while the plasma bubble is plotted by green iso-surfaces. Electrons injected into the bubble are accelerated to relativistic velocities by the longitudinal accelerating field, moving in a betatron-like oscillatory motion due to the transverse focusing field. X-ray betatron photons are emitted within a cone in the direction of electron momentum and propagate in front of the electron bunch.

### Nonlinear QED versus Liénard–Wiechert potentials

To check the validity of our approach we have compared the emission spectra of betatron radiation in full-scale 3D PIC models which simulate the LWFA using a nonlinear QED modified event generator with that of the radiation diagnostics using the Liénard–Wiechert potential. The results, obtained for the experimental conditions described in section “Methods”, are presented in Fig. 1b. The in situ synthetic radiation diagnostic is performed using Liénard–Wiechert potentials. The form factor that takes into account the discrete electron distribution associated with them is capable of quantifying the coherent and incoherent radiation with the transition at $\hbar \omega _{\textrm{coh}}$. Incoherent radiation is radiation whose wavelength is shorter than the distance between electrons, while coherent radiation is radiation whose wavelength is longer than the distance between electrons. With this form factor, the radiation with energy below $\hbar \omega _{\textrm{coh}}$, shown in Fig. 1b, can be calculated. The inclusion of the form factor is discussed in section “Methods”. The post-processing radiation diagnostic reproduces incoherent radiation. The calculation using the QED-modified event generator with radiation probability at the classical limit reproduces the spectrum down to the photon frequency that can be resolved by the computational domain grid. The results of the three approaches are in excellent agreement for $\hbar \omega \ge \hbar \omega _{\textrm{coh}}$.

The agreement between our QED approach and the classical Liénard–Wiechert potential approaches for the incoherent part of the x-ray spectrum indicates that our method is suitable for describing the betatron radiation in LWFA conditions. However, the unique characteristic of this method, that promotes its applicability, is the dramatic reduction of the computational cost. Specifically, the in situ synthetic radiation diagnostic run on 16 V100 GPUs over a wall-clock time of 217 h. The post-processing radiation diagnostic run for 87 wall-clock time hours on recording the trajectory on 16 k40 GPUs on the GPU node “island” of ARIS in the National HPC facility of the Greek Research and Technology Network (GRNET)^38^. An additional computational amount of time was demanded for the extraction of trajectories and the computation of the radiation spectrum. This time amount depends on the number of sample particles considered. The data collected amounted to 60 GB. However, the calculation using the QED-modified event generator recorded a striking reduction in computational time, which ran for approximately 10 h with 16 k40 GPUs. This outperformed the in situ diagnostic that runs on faster devices.

### Helium gas target

**Figure 2:**
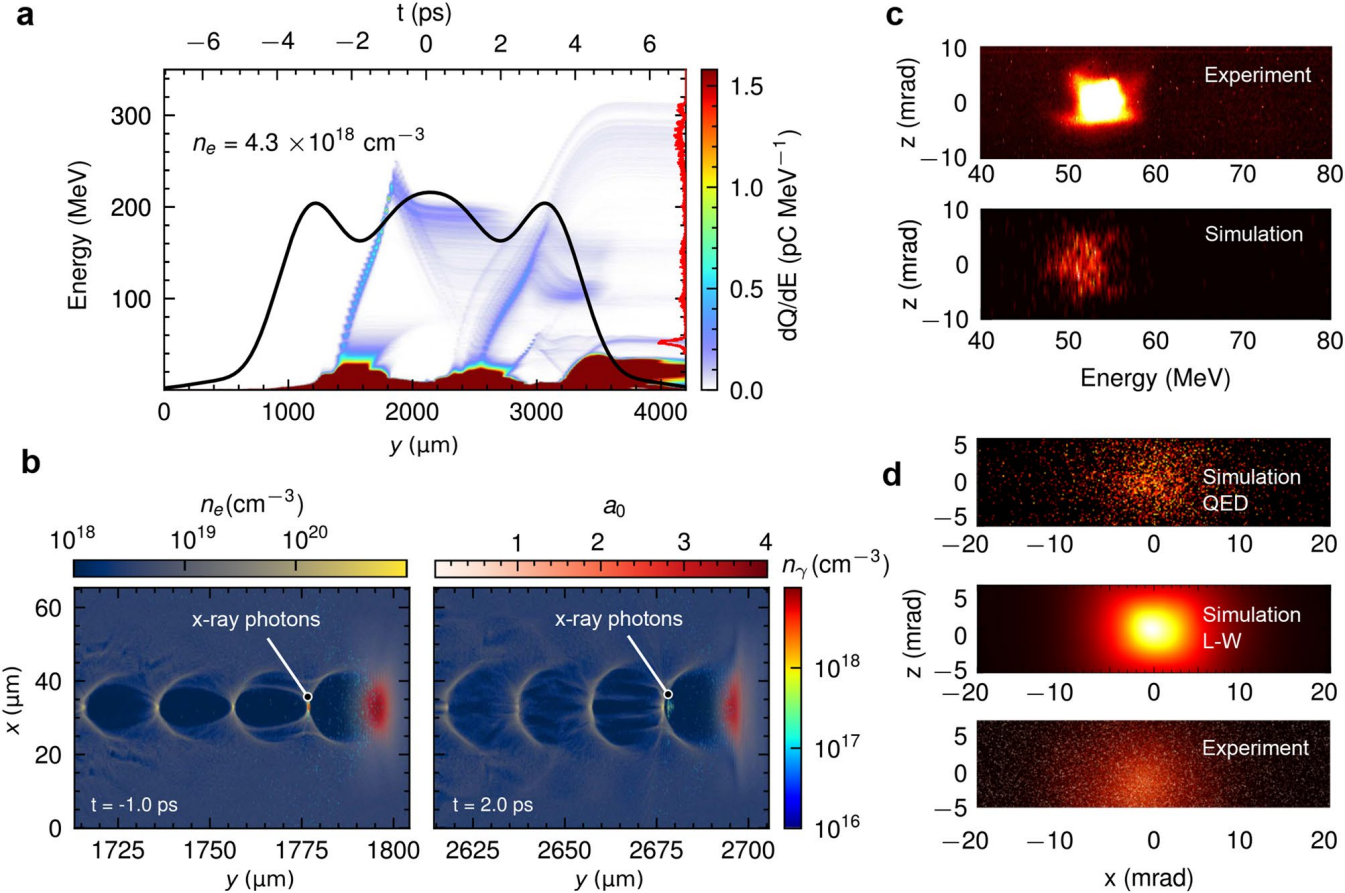
Laser-driven betatron radiation in 3 mm diameter nozzle helium gas jet. (**a**) Time evolution of the electron charge distribution over energy (electron spectrum) and its evolution over time along the laser propagation axis. The black solid line is the experimentally determined gas density profile with maximum electron density $n_e=4.3\times 10^{18} \, \textrm{cm}^{-3}$. The red line plot on the right vertical axis corresponds to the final electron spectrum at the exit of the gas jet. The top axis indicates the time of laser propagation and the laser is focused at $t=0$. (**b**) Snapshots of plasma density, laser envelope, and x-ray photon density maps at $t=-1.0\,\textrm{ps}$, and $t=2.0\,\textrm{ps}$. (**c**) Comparison between the simulated and the measured electron spectra. (**d**) Comparison between the simulated and the measured x-ray photon spectra.

The modified event generator with emission probability in the classical regime in a full-scale 3D PIC simulation allows for scrutinizing the betatron experiment fast and accurately. Simulations are performed with realistic experimental conditions, as described in the section “Methods”, to investigate betatron radiation for realistic laser-plasma dynamics. The laser is focused in the center of the jet (at $t=0$) with a measured density profile shown in Fig. 2a. The fully ionized helium gas with maximum plasma density $n_e = 4.3\times 10^{18} \,\textrm{cm}^{-3}$ is assumed. The laser wakefield operates in the bubble regime. According to the findings shown in Fig. 2a, the electrons are accelerated at the valleys of the plasma profile, following primarily a down-ramp injection mechanism. The first accelerated electrons originate from the high-density plasma at the rear of the bubble, as depicted in Fig. 2b at $t=-1\,\textrm{ps}$. The x-ray photons are emitted at the rear of the bubble. Then, the plasma bubble shrinks at the second peak of the profile and expands at the second down-ramp. Electrons are injected into the bubble at $t=2\,\textrm{ps}$. As shown in Fig. 2a, the final electron spectrum has only a very small amount of high energy electrons accelerated to $200-300 \, \textrm{MeV}$, while the lower energy electrons are seen to be self-injected into the plasma close to the area exiting the gas. Thus, the final most intense electron bunch is attributed to the late injections at the end of the nozzle with lower energy. The resulting electron bunch has energies between $50-55 \, \textrm{MeV}$, with the angular divergence of $\sim 9\,\textrm{mrad}$ at half width at half maximum (HWHM), which is consistent with the experimental result as shown in Fig. 2c. The x-ray divergence is $5 \, \textrm{mrad}$ HWHM horizontally and $5 \, \textrm{mrad}$ HWHM vertically, for the simulation calculated with the modified event generator and radiation diagnostics. The angular distribution and the divergence are also in very good agreement with the measured spectrum, as shown in Fig. 2d.

Next, we examine the relation between the observed electron spectrum and the corresponding betatron radiation, measured simultaneously on a shot-to-shot basis. The x-ray angular divergence in the wiggler regime, $\theta =1.33\times 10^{-10}\sqrt{n_e[\textrm{cm}^{-3}]/\gamma }r_\beta [\upmu \textrm{m}]$, predicts $7 \lesssim \theta \lesssim 33\, \textrm{mrad}$ with a betatron oscillation amplitude of $0.5 \lesssim r_\beta \lesssim 2.5 \,\upmu \textrm{m}$ for $\gamma \sim 97.84$. This is larger than the observed value. Moreover, the integrated electron charge is calculated to be $0.8-5\,\textrm{pC}$ for electron energies between $40-80\,\textrm{MeV}$, which is one order of magnitude smaller than the experimentally estimated value of $84-97\,\textrm{pC}$^39^. These estimations suggest that the observed electron bunch centered at $50-55 \, \textrm{MeV}$ is not the source of the observed betatron radiation. Instead, the x-ray emission is rather attributed to the betatron motion of the earlier electron injection which has been absorbed before exiting the gas jet.

### Ionization and Plasma wakefield

**Figure 3:**
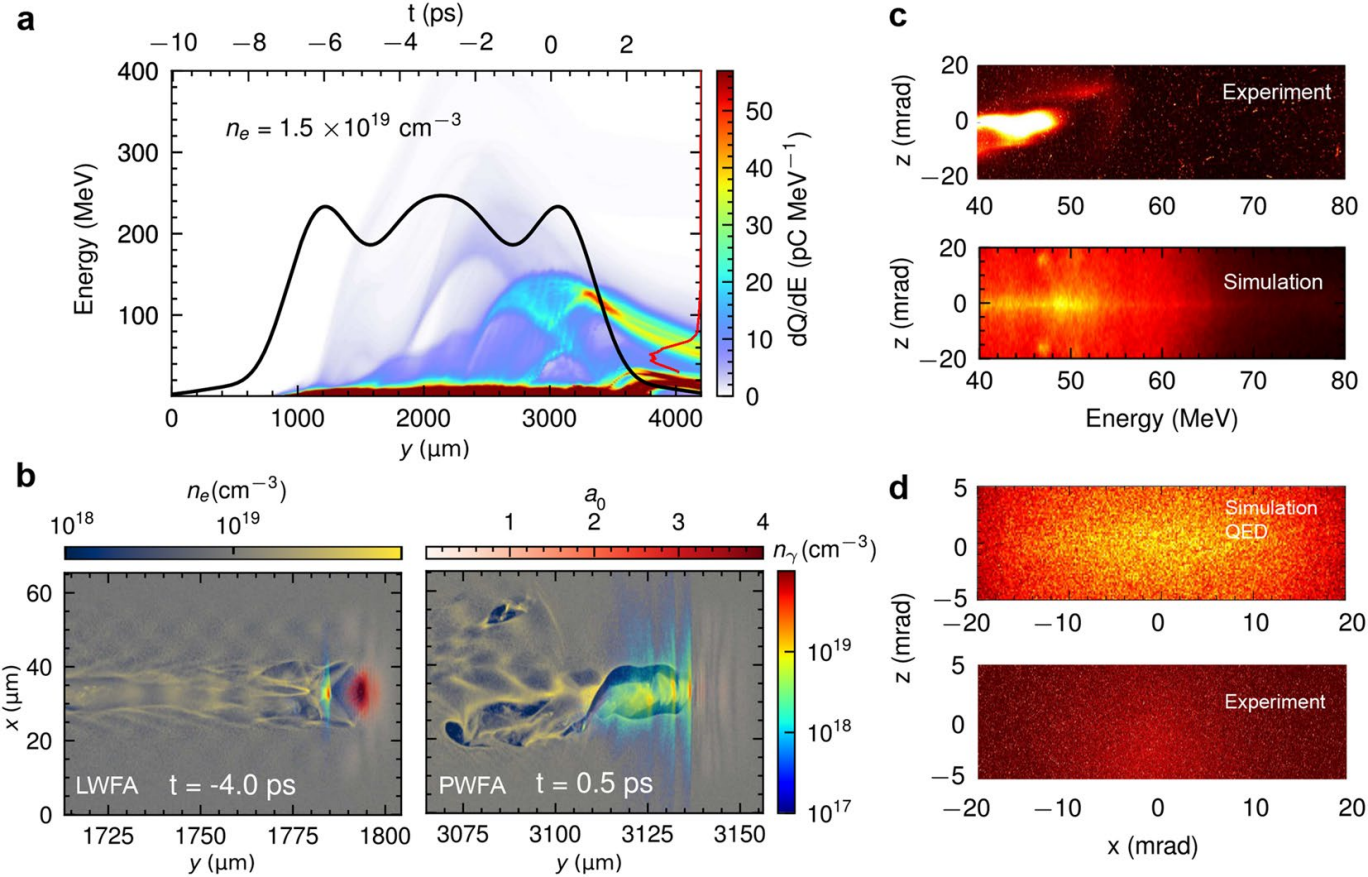
Laser-driven betatron radiation in 3 mm diameter nozzle nitrogen gas target. (**a**) Same as in Fig. 2a but for nitrogen. (**b**) Snapshots of plasma density, laser envelope, and x-ray photon density maps at $t=-4.0\,\textrm{ps}$, and $t=0.5\,\textrm{ps}$. The acceleration mechanism transfers from LWFA to PWFA. (**c**) Comparison between the simulated and the observed measured electron spectra. (**d**) Comparison between the simulated and the observed measured x-ray spectra.

The same simulations were performed using nitrogen gas with the laser focused at the end of the gas nozzle (at $t=0$). The leading edge of the intense laser pulse is strong enough to ionize five outer electrons of a nitrogen atom^39^. The electron density of the same profile with helium is now $n_e = 1.5\times 10^{19} \,\textrm{cm}^{-3}$ as shown in Fig. 3a. Electrons are mostly injected at the valleys of the plasma profiles, similar to the case of helium, but with relatively broad energy spectra and low electron charge. This corresponds to the laser-driven wakefield acceleration shown in Fig. 3b. The bubble radius is about the pulse duration at $t=-4\,\textrm{ps}$. The injected electrons propagate with the photon beam. The laser energy is depleted after the propagation distance of, $L_{depl}\sim 0.87\,\textrm{mm}$, and the intensity is $a_0 \ll 1$. The wakefield acceleration is now passing from the laser-driven to the beam-driven regime as shown in Fig. 3b at $t=0.5\,\textrm{ps}$. A higher charge is also observed at the exit of the gas jet as shown in Fig. 3a. The structure of the wakefield is highly unstable; however, the betatron emission continues. The electron bunch exiting the gas target has a broad spectrum up to $\sim 50\,\textrm{MeV}$, with divergence $\lesssim 5 \, \textrm{mrad}$ HWHM for both simulation and experiment as shown in Fig. 3c. Figure 3d shows the x-ray angular distribution with the divergence of $20\,\textrm{mrad}$ HWHM horizontally, and $20\,\textrm{mrad}$ HWHM vertically. Regardless of laser or beam-driven wakefield acceleration, the radiated spectrum is synchrotron-like with critical energy $\hbar \omega _{\textrm{c}}=58.6\,\textrm{keV}$. The total simulation time for nitrogen gas using the modified event generator is 16 h on 16 k40 GPUs, which is $33\times$ faster than the in situ synthetic radiation diagnostic on 16 V100 GPUs. Due to the increased number of electrons the data recorded for post-processing radiation diagnostic overcomes the storage limit of $10 \,\textrm{TB}$ for further trajectory tracking.

## Discussion

**Figure 4:**
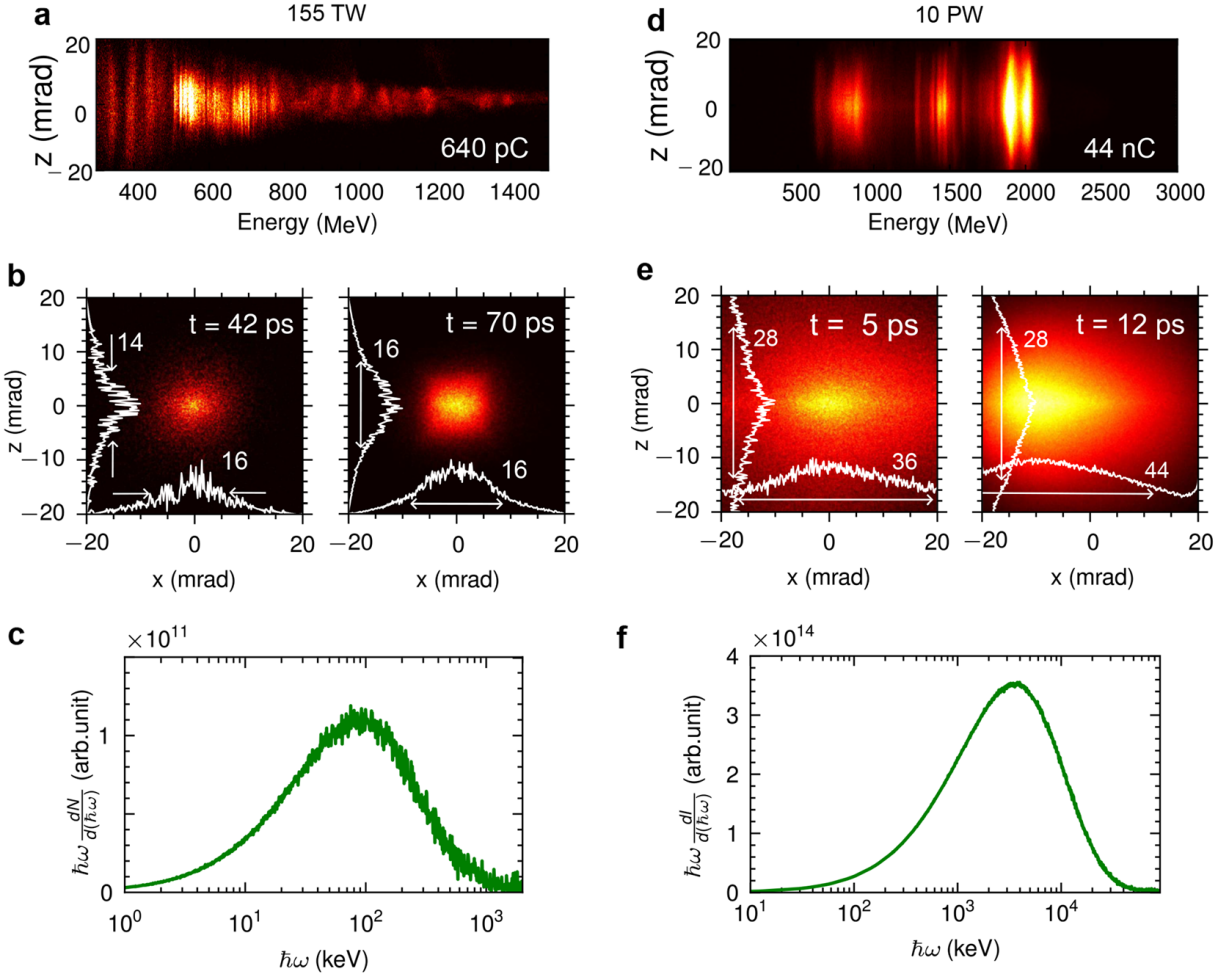
picongpu simulations of laser-driven betatron radiation for different laser power and acceleration length. (**a**–**c**) Simulated results for electron acceleration along $2.0\,\textrm{cm}$ gas cell. The laser power on target is $155\,\textrm{TW}$. (**a**) Electron spectrum at the exit of the gas cell ($L=2.2\,\textrm{cm}$) with calculated charge of $640\,\textrm{pC}$. (**b**) X-ray spectra at $t=42\,\textrm{ps}$ and $t=70\,\textrm{ps}$. (**c**) X-ray energy spectra with $\hbar \omega _{\textrm{c}}= 310\, \textrm{keV}$. (**d**–**f**) Simulated results for $10 \,\textrm{PW}$ peak power laser pulse in 3 mm diameter nozzle gas jet. The setup is similar to Fig. 2 Gaussian plasma density profile. (**d**) Electron spectrum with a calculated charge of $44\,\textrm{nC}$. (**e**) X-ray spectra at $t=5\,\textrm{ps}$ and $t=12\,\textrm{ps}$. (**c**) X-ray energy spectra with $\hbar \omega _{\textrm{c}}= 13.7\, \textrm{MeV}$. On-axis lineouts in (**b**) and (**c**) are included with the divergence at FWHM.

To demonstrate the robustness of the betatron simulation using the modified event generator, we performed additional simulations for a longer acceleration length and a multi-petawatt laser system. We performed a simulation of LWFA for electron acceleration in a $2\,\textrm{cm}$ gas cell. A $55\,\textrm{fs}$ duration laser pulse with $a_0=2$ is focused on a spot size of $40\,\upmu \textrm{m}$. The laser power on target is $155\,\textrm{TW}$. The plasma peak density is $n_e=2.0 \times 10^{18} \,\textrm{cm}^{-3}$. The accelerated electron at the exit of the gas cell ($L=2.2\,\textrm{cm}$) has energy up to $800\,\textrm{MeV}$ and a calculated charge of $640\,\textrm{pC}$ as shown in Fig. 4a. The x-ray beam divergences in Fig. 4b are rather consistent throughout the acceleration process with $\sim 16\,\textrm{mrad}$ FWHM horizontally, and vertically. The x-ray spectrum in Fig. 4c exhibits a peak at $90\,\textrm{keV}$ and critical energy at $310\,\textrm{keV}$. The simulation closely reproduces the experimental results of strongly resonant betatron radiation in Ref.^6^. The total simulation time is 23.2 h on 16 k40 GPUs. For an even longer gas cell, i.e. $10\,\textrm{cm}$ capillary discharge, the dispersionless Maxwell’s solver coupled with the Lorentz boosted frame may be required.

With a higher-power laser, a high-energy bunch with a charge of tens-nC is expected. This is possible with the $10 \, \textrm{PW}$ laser facility, where the pulse is compressed to $\tau \lesssim 25 \,\textrm{fs}$, and the wakefield is running in the bubble regime. The required plasma density is $n_e\sim 10^{19} \,\textrm{cm}^{-3}$, with a laser energy of $266 \,\textrm{J}$ focused on $w_0=10\,\upmu \textrm{m}$. The laser has an intensity of $6\times 10^{21} \,\mathrm {W \, cm}^{-2}$ ($a_0=53$). Such a high-energy and high-charge bunch would provide a focusing gradient in the cavity higher than $1\,\mathrm {TV \, m}^{-1}$, resulting in betatron $\gamma$-ray emission in the range of $\gtrsim 10\,\textrm{MeV}$. Figure 4d shows the electron spectra with a peak energy of $2 \,\textrm{GeV}$, divergence $\lesssim 20 \,\textrm{mrad}$, and a calculated charge of $44\,\textrm{nC}$. These results closely resemble the betatron radiation generation in the QED regime reported in Ref.^40^. At $t=12\,\textrm{ps}$, the laser pulse exits the gas jet and undergoes beam spreading in the vacuum. The divergence is rather large ($\sim 28-44 \,\textrm{mrad}$), although the electron energy is in the GeV range as shown in Fig. 4e. The spectrum exhibits critical energy at $13.7\,\textrm{MeV}$ (Fig. 4f). The total simulation time is 30.4 h on 32 k40 GPUs. The calculation of the betatron emission spectrum for tens-of-nC electrons with in situ or postprocessing radiation diagnostic becomes extremely challenging, even if the acceleration length is just a few millimeters.

### Conclusion

In summary, our study demonstrates the applicability of the non-linear QED approach running at the classical regime to betatron radiation description in laser-wakefield acceleration. Our proposed QED approach reproduces the LWFA betatron-type experimental findings for various gas targets and laser power regimes. We quantitatively demonstrate a significant reduction in computational demands compared to using the Liénard–Wiechert potential method, either in situ or post-processing. This, in turn, reduces the difficulty of reproducing experimental results, which frequently necessitates multiple runs and parameter scanning. Our research provides a more efficient framework for designing betatron radiation experiments with various schemes–from ionization injection to multiple-stage acceleration, especially using higher-power lasers.

## Methods

### Experiment

The experiments were performed at the Institute of Plasma Physics and Lasers-IPPL of the Hellenic Mediterranean University Centre of Research & Innovation using the 45 TW fs laser system “Zeus”, that delivers pulses with a maximum energy of 1.3 J, central wavelength at $\sim 800$ nm and duration of 25 fs, at a repetition rate up to 10 Hz^41^. A secondary beam is delivered by the laser system at an energy of 10 mJ and pulse duration of 25 fs, that is used for probing the plasma channel at controlled time delays. The experimental setup has been described in detail in Ref.^39^ and related reports^42–44^, and only a brief description will be given here for completeness purposes.

The laser beam is focused on the gas jet target by a 1 m focal length off-axis parabolic mirror. The pulsed gas jet flow was shaped by an electromagnetic valve having a 3 mm diameter nozzle, synchronized with the laser repetition rate. The gas density profiles were determined using the secondary laser beam in a Nomarski-type interferometric setup and the use of density reconstruction algorithm. The gas density measurements were used for the estimation of the plasma density taking into consideration the degree of ionization for the gases used.

The generated x-rays were recorded using a vacuum-installed 16-bit x-ray CCD camera with a sensor having 2048$\times$ 512 pixels. A 10 $\mu$m thick Al foil was placed in front of the x-ray camera to filter the IR laser beam, as well as other secondary light sources in the EUV region. The relativistic electrons were recorded using a magnetic spectrometer consisting of two permanent magnetic plates placed in parallel at a separation distance of 1 cm, thus resulting in a homogeneous magnetic field, measured 0.4 T. The relativistic electron beam driven by the magnetic field impinged on a rectangular scintillating screen and the emitted light was imaged by a lens onto a CCD camera. The relativistic electron spectra were obtained from the CCD images according to relativistic electron orbit calculations. The secondary relativistic electrons and x-rays, emitted along the laser beam propagation axis, are detected simultaneously on a shot-to-shot basis.

### Particle-in-cell simulation

The simulations of LWFA were performed using the 3D PIC code picongpu version 0.5.0. A 3D computational domain of size $65.5 \times 91.2 \times 65.5 \,\upmu \textrm{m}^3$ is descritized by $256 \times 2280 \times 256$ cells and a time-step $\Delta t = 1.28 \times 10^{-16} \,\textrm{s}$ is set for the simulation.

The gas density profile, which was determined experimentally, is modeled by using Fourier series fitting to the measured profile^45^. The gas density is $2.15 \times 10^{18} \,\textrm{cm}^{-3}$. The helium is assumed to be fully ionized and nitrogen is 5 times pre-ionized with 2 macroparticles per cell. Further ionization of nitrogen ions is modeled using the Ammosov-Delone-Krainov (ADK) model^46^ for linearly polarized laser field and barrier suppression ionization (BSI)^47^. The intensity of the laser pulses focused at the target area was experimentally determined to $7\times 10^{18} \,\mathrm {W/cm}^2$ ($a_0=1.8$) assuming a spatial and temporal Gaussian profile. The laser pulse propagates in the y-direction and polarizes in the x-direction. The moving window traced the plasma bubble evolution and electron injection along the laser propagation direction.

The evolution of electric and magnetic fields was calculated by Lehe’s solver to reduce the growth of the beam emittance of accelerated electron bunches and reduce the effect of numerical Cherenkov radiation^4^. The electron macroparticle has a triangular shape and is propagated by Vay’s pusher^48^. The photons are propagated at the speed of light in the direction of the electron. The current was calculated using Esirkepov’s current deposition scheme^49^.

The simulation in a gas cell was performed in a computational domain of size $80 \times 105 \times 80 \,\upmu \textrm{m}^3$ discretized by $200 \times 2625 \times 200$ cells. The time step is $\Delta t = 1.28 \times 10^{-16} \,\textrm{s}$. The pre-ionized plasma with density $2.0 \times 10^{18} \,\textrm{cm}^{-3}$ is used. The plasma has a Gaussian up-ramp profile of $100 \,\upmu \textrm{m}$, followed by $2 \,\mathrm {c~m}$ plateau, and then a Gaussian down-ramp of $100 \,\upmu \textrm{m}$. One macroparticle-per-cell is used. A laser pulse of $55\,\textrm{fs}$ duration with $a_0=2$ is focused on a spot size of $40\,\upmu \textrm{m}$ at the beginning of the up-ramp. The laser pulse in Lehe’s solver propagates with numerical group velocity $v_g \gtrsim c$ and outruns the moving window after a certain distance of propagation. The moving window speed is set to $v_g=1.001c$ to secure the monitoring of the laser pulse propagation within the simulation domain.

The simulation of the $10\,\textrm{PW}$ laser was performed in a simulation domain of size $100 \times 109.2 \times 100 \,\upmu \textrm{m}^3$ discretized by $304 \times 2730 \times 300$ cells. The time step is $\Delta t = 1.28 \times 10^{-16} \,\textrm{s}$. Pre-ionized plasma with density $1 \times 10^{19} \,\textrm{cm}^{-3}$ is used. The plasma has a super-Gaussian up-ramp profile of $500 \,\upmu \textrm{m}$, followed by $1600 \,\upmu \textrm{m}$ plateau, and then a super-Gaussian down-ramp of $500 \,\upmu \textrm{m}$. Two macroparticles per cell are used. A $25\,\textrm{fs}$ duration laser pulse with $a_0=53$ is focused on a spot size of $11.8\,\upmu \textrm{m}$ ($w_0=10\,\upmu \textrm{m}$) at the beginning of the plateau.

Particle calorimeters are placed at infinite distances in the $+y$-direction to collect the energy of electrons and photons. The electron calorimeter has $128 \times 128$ resolution for the $10^\circ \times 10^\circ$ opening angle in the *x* and $z-$ direction. Meanwhile, the photon calorimeter has $256 \times 256$ resolution for $2.3^\circ \times 2.3^\circ$ opening angle in *x* and $z-$direction. Both electron and photon energies are sampled over 2048 grid points in the logarithmic scale.

### In situ synthetic radiation diagnostic

Betatron radiation was calculated using the radiation plugin implemented in the code^20–22^. This plugin calculates the Liénard–Wiechert potential for each electron macroparticle at every timestep:

2 $$\begin{aligned} \frac{d^2I}{d\omega d\Omega }=\frac{e^2}{16\pi ^3\epsilon _0 c} \times \left|\sum _{j = 1}^{N_e} \int _{-\infty }^{\infty } \frac{{\textbf{n}}\times [({\textbf{n}}-\varvec{\beta }_j)\times \dot{\varvec{\beta } } ]}{1- \varvec{\beta }_j\cdot {\textbf{n}} } e^{i\omega [t-{\textbf{n}}\cdot {\textbf{r}}_j/c ]} dt\right|^2 \end{aligned}$$

where, ${\textbf{r}}_j$, $\varvec{\beta }_j$, and $\dot{\varvec{\beta }}$ are the position, normalized velocity, and normalized acceleration of the electron *j*. To reduce the simulation cost, the radiation emission is computed only for electrons with energy above $50 \,\textrm{MeV}$ for helium and $25 \,\textrm{MeV}$ for nitrogen. The simulated radiation energy ranges from 0.0001 to 100 keV and is sampled over 2048 grid points in the logarithmic scale. The simulation resulted in radiation emitted within $\theta =\pm 10^\circ$ at $\phi =0^\circ$. The 64-bit precision is used for special operations. The PIC simulation uses macroparticles to represent an ensemble of electrons with a continuous charge distribution. To calculate the coherent and incoherent contribution accurately, the shape of the macroparticle must be taken into account by associating it with a form factor $F^2(\omega ) = N + (N^2 - N) \cdot ({\mathcal {F}}(\rho (x)))^2$, where *N* is the number of electrons modelled by a macroparticle and ${\mathcal {F}}(\rho )$ is the Fourier transform of the charge distribution $\rho$. The first term represents the incoherent radiation component and the second is the coherent part due to interference effects between the *N* electrons. The Gauss spherical form factor for macroparticles was utilized to account for the discrete nature of electrons represented by the macroparticle, which can correctly include both coherent and incoherent radiation. The boundary between these two regions is $\hbar \omega _{\textrm{coh}} = \hbar c n_e^{1/3} = 0.023 \,\textrm{keV}$ for helium gas. The frequency distribution of the total energy emitted can be determined by integration over the angles: $dI/d\omega =2\pi \int (d^2I/d\omega d\Omega ) \cos \theta d\theta$. The photon energy distribution is $dN/d\omega = (1/\omega ) dI/d\omega$.

**Figure 5:**
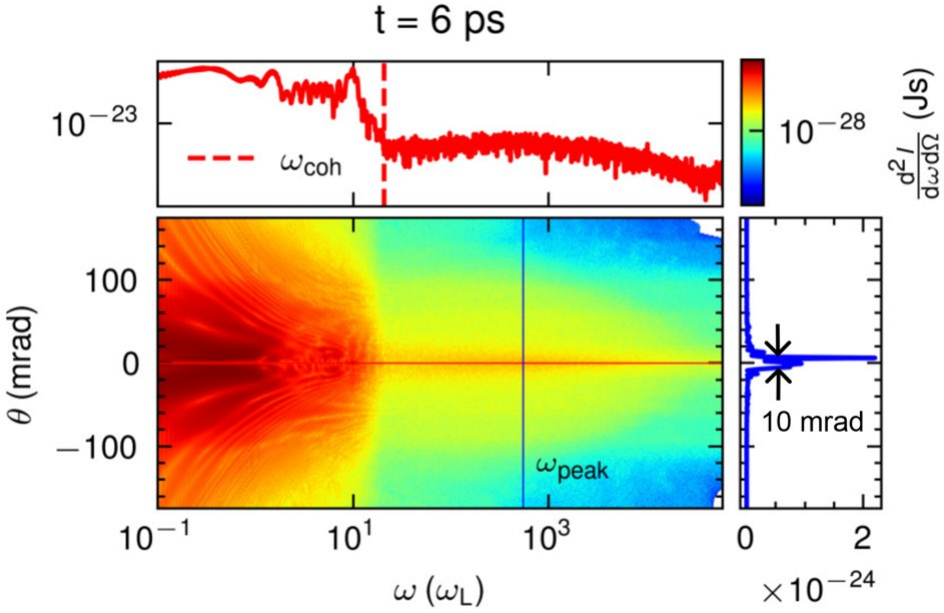
Emitted x-ray energy per unit frequency and unit solid angle as a function of the frequency and the observation angle. Transition between coherent and incoherent radiation is at $\omega _{\textrm{coh}}=4.89\times 10^{16}\,\textrm{s}^{-1}=21\omega _{\textrm{L}}$. The graphs on the top and right panels are the lineouts along frequency and angle, respectively. The FWHM of divergence is estimated to be within $10\,\textrm{mrad}$.

To get further insight into the physics of coherent and incoherent radiation emission we show the frequencies-angular map of x-ray radiation calculated using Liénard–Wiechert potential in Fig. 5, corresponding to the same parameters as in Fig. 1. The map shows the distinct separation of coherent and incoherent radiation at $\omega _{\textrm{coh}}=4.89\times 10^{16}\,\textrm{s}^{-1}=21\omega _{\textrm{L}}$, judging by their angular dependence. The incoherent part is emitted along a very narrow cone by the betatron motion with the FWHM divergence estimated to be within $10\,\textrm{mrad}$. The emission below $\omega _{\textrm{coh}}$ could be attributed to the coherent emission due to acceleration over short distances compared to the radiated emission^50^ or electro-optic shocks^51^ during the injection. Both scenarios exhibit emission with a wide angular distribution compared to the betatron radiation.

### Post-processing radiation diagnostic

A separate simulation was performed to record electron trajectories with energy greater than 50 MeV for every two time steps. These trajectories were used to calculate the radiation emission using Eq. (2) in postprocessing with the SynchRad Python package^52^.

### QED: modified event generator

The photon emission probability rate in the constant cross-background field is described by^25^

3 $$\begin{aligned} dW_{\textrm{QED}} = \frac{\alpha mc^2}{\sqrt{3}\pi \hbar \gamma } \left[ \left( 1-\xi + \frac{1}{1-\xi } \right) K_{2/3}(\delta ) - \int _\delta ^\infty K_{1/3}(s)ds \right] d\xi \end{aligned}$$

where, $\delta =2\xi /(3\chi [1-\xi ])$, $\xi =\hbar \omega /\gamma mc^2$, and $K_\nu (x)$ is the modified Bessel function. The Lorentz invariant parameter $\chi$ determines the transition between the classical and quantum description of radiation emission

4 $$\begin{aligned} \chi = \frac{e\hbar }{m^3 c^4} \sqrt{\left( \frac{\varepsilon {\textbf{E}}}{c} + {\textbf{p}} \times {\textbf{H}}\right) ^2 - (\mathbf {p\cdot E})^2}. \end{aligned}$$

Here, $\varepsilon =\gamma mc^2$ and ${\textbf{p}}$ are the energy and momentum of the electron, respectively; ${\textbf{E}}$ and ${\textbf{H}}$ are the local electric and magnetic fields experienced by the electron, *m* is the rest mass of the electron, $\hbar$ is the reduced Planck constant and *c* is the speed of light in vacuum. For $\chi \ll 1$, the radiated energy is small compared to the electron energy, and the radiation can be treated classically. For $\chi \gtrsim 1$, the electron radiates a significant amount of its kinetic energy into photon emission, and the electron recoil causes a change in the trajectory.

**Figure 6:**
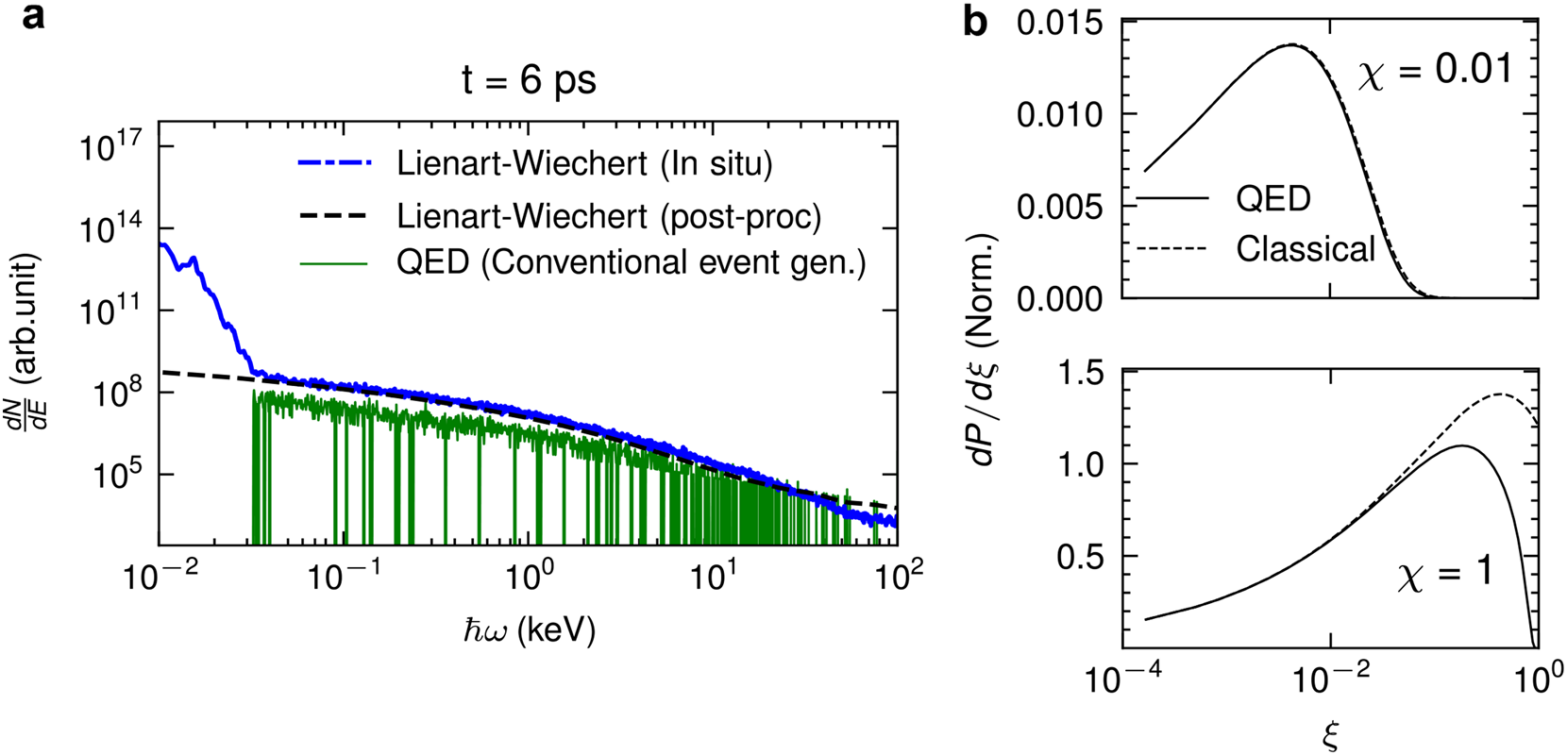
(**a**) The simulated betatron x-ray spectra calculated with in-situ synthetic radiation diagnostic (dash-dotted line), and post-processing (dashed line) radiation diagnostic with Liénard–Wiechert potentials, and conventional event generator in the classical limit (solid line). (**b**) Normalized emission spectra at $\chi = 0.01$ and $\chi = 1$. The QED spectra (solid line) are based on Eq. (5), and the classical spectra (dashed line) are based on Eq. (6).

The spectrum of emissions is

5 $$\begin{aligned} \frac{dP_{\textrm{QED}}}{d(\hbar \omega )}= & {} \xi \frac{dW_{\textrm{QED}}}{d\xi } \nonumber \\= & {} \frac{\alpha mc^2 \xi }{\sqrt{3}\pi \hbar \gamma } \left[ \left( 1-\xi + \frac{1}{1-\xi } \right) K_{2/3}(\delta ) - \int _\delta ^\infty K_{1/3}(s)ds \right] \end{aligned}$$

and reduces to the classical synchrotron emission for $\xi , \chi<<1$:

6 $$\begin{aligned} dP_{\textrm{cl}} = \frac{e^2\omega _c}{\sqrt{3}\pi c\gamma ^2} \frac{\omega }{\omega _c} \int _\delta ^\infty K_{5/3}(s)ds \end{aligned}$$

where, now $\delta =2\xi /3\chi =\omega /\omega _c$, and $\hbar \omega _c \sim 1.5\chi \gamma mc^2$. For $\chi \lesssim 0.01$, the classical emission spectrum has no difference from the QED spectrum, while for $\chi \gtrsim 1$ the classical spectrum overestimates the emission at $\xi =1$, as shown in Fig. 6b.

Event generators determine the emission of a photon and its energy^26,27^. Two random variables $\{r_1, r_2\}\in [0,1]$ are generated with uniform probability. A photon with energy $r_1\gamma mc^2$ is emitted if $r_2<\text {Pr}(r_1)$, where

7 $$\begin{aligned} \text {Pr}(\xi )= & {} \frac{\partial P_{\textrm{QED}}}{\partial (\hbar \omega )} \frac{\partial (\hbar \omega )}{\partial \xi } \frac{1}{\hbar \omega } \Delta t \nonumber \\= & {} \frac{\alpha mc^2}{\sqrt{3}\pi \hbar \gamma } \left[ \left( 1-\xi + \frac{1}{1-\xi } \right) K_{2/3}(\delta ) - \int _\delta ^\infty K_{1/3}(s)ds \right] \Delta t \end{aligned}$$

is the probability of emission of one photon in a single time step, $\Delta t$. The time step is required to be small such that $\text {Pr}(\xi )<1$ is always met.

However, the probability of photon emission encounters the infrared divergence with the emission of a low-energy photon. In the limit $\xi \ll 1$, $\text {Pr}(\xi )\approx \Delta t (\alpha mc^2/\hbar )\times 0.52 \gamma ^{-1} \xi ^{-2/3} \chi ^{2/3}$ and $\text {Pr}(0) > 1$. Thus, the low-energy photon emission cannot be handled by the event generator. As depicted in Fig. 6a, even if a maximum threshold probability of 0.4 is set, it would lead to an underestimation of the photon spectrum in the low-energy range. Typically, a cutoff point is applied at the photon energy $\hbar \omega <2mc^2$ to exclude the divergence and not to affect subsequent pair production. However, the photon energy for a typical betatron radiation is below this value and thus, the cutoff would result in no photon emission. This restriction seems to make the QED approach unsuitable for the description of betatron radiation. To overcome this problem and maintain the event generator concept within the QED description for the low-energy photon emission, a modified event generator is introduced with a scaled photon emission probability

8 $$\begin{aligned} \text {Pr}_m(r_1) {:}{=}\frac{\partial f_m(r_1)}{\partial r_1}\text {Pr}[f_m(r_1)], \end{aligned}$$

where $f_m(r_1)$ is a function chosen to remove the infrared divergence. The divergence is eliminated with $f_m(r_1)=r_1^3$, and $\text {Pr}_m(0) < 1$. The simulation time step is chosen such that $\text {Pr}_m(r_1) \ll 1$. This modified event generator is implemented in the picongpu code^53^.

The time step limit for betatron photon emission after considering the modified event generator is $\Delta t \ll \chi ^{-2/3}\gamma \times 1.0\times 10^{-19} \, \textrm{s}$. For $\chi \sim O (10^{-3})$, and $\gamma \sim O (10^2)$, then $\Delta t \ll 1.0\times 10^{-15} \, \textrm{s}$, which is satisfied in this simulation. The modified event generator only considers the photon emission with frequencies that cannot be resolved by the computational grid.

## Data Availability

The datasets used and/or analyzed during the current study are available from the corresponding author upon reasonable request.
